# A threshold of endogenous stress is required to engage cellular response to protect against mutagenesis

**DOI:** 10.1038/srep29412

**Published:** 2016-07-11

**Authors:** Yannick Saintigny, François Chevalier, Anne Bravard, Elodie Dardillac, David Laurent, Sonia Hem, Jordane Dépagne, J. Pablo Radicella, Bernard S. Lopez

**Affiliations:** 1 Institute of Cellular and Molecular Radiobiology –Commissariat à l’Energie Atomique et aux Energies Alternatives, Fontenay aux Roses, F-92265, France; 2 UMR967 INSERM/CEA/Universités Paris Diderot et Paris Saclay, Fontenay aux Roses, F-92265, France; 3 UMR 8200 CNRS, Institut de cancérologie Gustave Roussy, Université Paris-Saclay, équipe labélisée par la Ligue bationale contre le Cancer “LIGUE 2014”, Villejuif, F-94805, France; 4 Plateforme de spectrométrie de masse protéomique – MSPP, Biochimie et physiologie moléculaire des plantes, CNRS, INRA, Montpellier Supagro, Univ. Montpellier, 34060 Montpellier, France

## Abstract

Endogenous stress represents a major source of genome instability, but is in essence difficult to apprehend. Incorporation of labeled radionuclides into DNA constitutes a tractable model to analyze cellular responses to endogenous attacks. Here we show that incorporation of [^3^H]thymidine into CHO cells generates oxidative-induced mutagenesis, but, with a peak at low doses. Proteomic analysis showed that the cellular response differs between low and high levels of endogenous stress. In particular, these results confirmed the involvement of proteins implicated in redox homeostasis and DNA damage signaling pathways. Induced-mutagenesis was abolished by the anti-oxidant N-acetyl cysteine and plateaued, at high doses, upon exposure to L-buthionine sulfoximine, which represses cellular detoxification. The [^3^H]thymidine-induced mutation spectrum revealed mostly base substitutions, exhibiting a signature specific for low doses (GC > CG and AT > CG). Consistently, the enzymatic activity of the base excision repair protein APE-1 is induced at only medium or high doses. Collectively, the data reveal that a threshold of endogenous stress must be reached to trigger cellular detoxification and DNA repair programs; below this threshold, the consequences of endogenous stress escape cellular surveillance, leading to high levels of mutagenesis. Therefore, low doses of endogenous local stress can jeopardize genome integrity more efficiently than higher doses.

Faithful and accurate transmission of genetic information through successive cell divisions requires the precise control of the DNA damage response (DDR), which coordinates a network of pathways protecting against genetic instability. Nonetheless, genetic variability is required in physiological processes, such as meiosis or the establishment of the immune repertoire, and is also a driving force for genome evolution. Therefore, the balance between genome diversity and stability must be tightly regulated to allow variability while preventing the accumulation of deleterious genetic modifications.

Cells are continuously exposed to exogenous as well as endogenous stresses that jeopardize genome integrity. Particularly, endogenous stress represents a significant biological phenomenon because cells are chronically exposed to it throughout their lifespan, constituting a major source of genome instability that fuels cancer development or senescence. However, endogenous stress is in essence difficult to apprehend.

Incorporation of labeled radionuclides into DNA results in internal *in situ* self-irradiation, thus constituting a tractable model of exposure to endogenous attack that allows analysis of cell responses to such endogenous stresses. Validating this approach, cells respond differently to internal radioactive contamination (endogenous stress) compared with external radiation^1^. Moreover, the radionuclide incorporation model facilitates the detection and analysis of the impact of lower doses compared to external γ-rays. Indeed, in an apparent paradox, the risk of damage increases when the energy emitted decreases since, when using radionuclides that incorporate into the DNA most of the energy is locally deposited into the nucleus by low-energy compounds, such as tritium^1^,^2^. Given its low disintegration energy, tritium’s biological effects do not occur from external exposure but from integration of organically bound tritium (OBT) into tissue^3^. In a previous study using labeled nucleoside [^3^H]thymidine, which is incorporated into DNA, we established a model to analyze the genetic impact of endogenous self-irradiation of DNA by a low energy-emitting source, such as tritium. We have demonstrated that low doses of incorporated [^3^H]thymidine, which are barely toxic, induce DNA double strand breaks and stimulate homologous recombination (HR) in a linear dose-response manner, reaching a plateau between 1 and 2 Gy/nucleus (>70% viability). In contrast, external γ-rays behave differently, and 4 to 6 Gy (>10% viability) are required to detect rare HR events^1^. These findings highlights the differences between external and internal (endogenous) exposure to radiation. Moreover, reactive oxygen species (ROS) resulting from endogenous metabolism is a major source of endogenous stress. Given that radiation generates ROS through water radiolysis, incorporation of labeled radionuclides constitutes a model for the production of local endogenous oxidative stress. Therefore, cellular responses to internal exposure should mimic many aspects of the response to endogenous stress.

To examine the genetic impact of endogenous exposure to genotoxic insults, we analyzed point mutations induced by [^3^H]thymidine incorporation into the genome. We demonstrate that [^3^H]thymidine generates oxidative stress and oxidative damage-induced mutagenesis. Strikingly, [^3^H]thymidine induces a peak of mutagenesis in a nonlinear dose-response curve at low doses but not at high doses. The peak of mutagenesis is explained by the fact that low doses escape cellular surveillance. However, when a threshold is achieved, the cell response induces both detoxification of ROS and induction of DNA repair enzymes specialized in oxidative DNA damage, thus decreasing the mutagenic outcome.

## Results

### Labeling of CHO cells with [^3^H]thymidine

**Figure 1:**
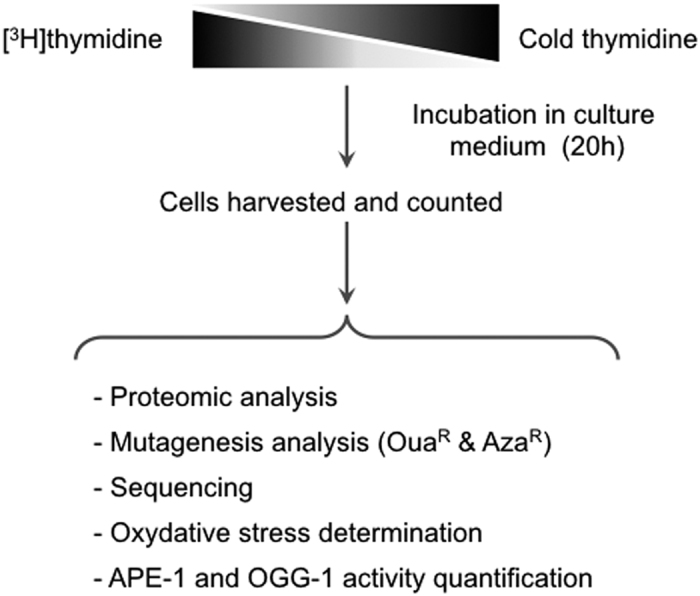
Experimental design. Cells were cultured in the presence of different specific activities of [^3^H]thymidine. The final concentration of thymidine was maintained constant supplying cold thymidine. After 20 h of incubation, when incorporation reached a plateau, greater than 95% of cells contained labeled nucleotides. Incorporated radioactivity was counted in the trichloroacetic acid (TCA) precipitate. Mutagenesis frequency, DNA sequence, ROS, antioxidant activities, and proteomics were subsequently measured.

CHO-K1 hamster cells lines were cultured in the presence of different specific activities of labeled [^3^H]thymidine^1^. These cell lines were chosen because the impact of [^3^H]thymidine incorporation on DNA double strand break repair has been extensively analyzed in this background^1^ and mutagenesis can be measured at different loci. All experiments were performed using a final concentration of thymidine that was maintained as a constant by supplying cold thymidine. Radioactivity incorporated into the DNA was counted in trichloroacetic acid (TCA) precipitates 20 hours after exposure to [^3^H]thymidine, i.e., when incorporation plateaued and greater than 95% of cells contained labeled nucleotides. Following cell counting, viability (colony-forming efficiency), protein extraction, oxidative stress determination and mutagenesis frequencies were assessed (Fig. 1). Doses are expressed as the amount of Grays (Gy) delivered into the cell nucleus during 20 hours^1^.

### Proteomic analysis of [^3^H]thymidine incorporation

We first explored the possibility of a difference in the cell response between low and high doses of incorporated [^3^H]thymidine without *a priori* exclusion of any factors. To do so, a gel-based proteomic approach was performed to study the proteome of cells upon [^3^H]thymidine incorporation, with a specific focus on nuclear proteins^4^,^5^,^6^.

**Figure 2:**
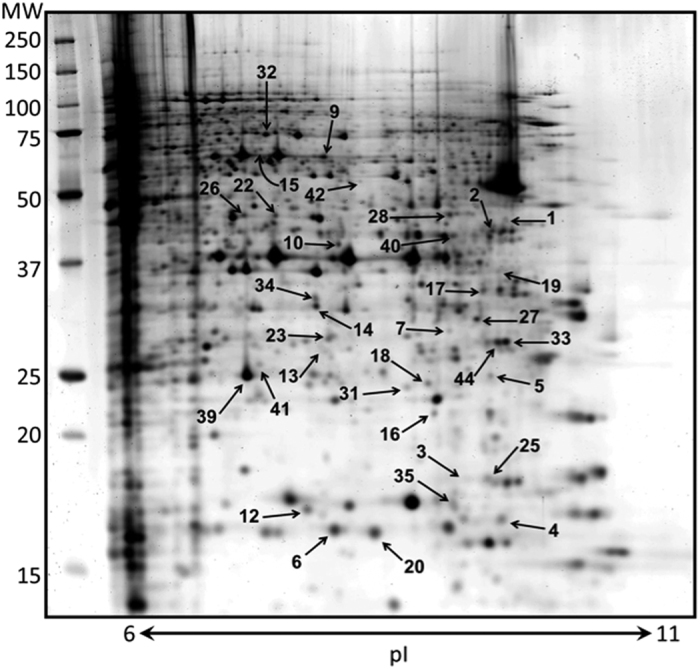
[^3^H]thymidine incorporation induces proteome modifications in CHO cell nuclear proteins. A typical two-dimensional electrophoresis image of CHO cell nuclear proteins is presented using 100 μg of soluble nuclear proteins. Proteins were visualized by Lava purple staining. Numbered spots were significantly increased or decreased in protein exacts from CHO cells upon [^3^H]thymidine incorporation. Proteins were identified by mass spectrometry (Table 1, supplementary data S1). Gene ontology of each protein was analyzed (Supplementary data S2), and the expression pattern of three representative proteins upon [^3^H]thymidine incorporation is presented in Fig. 3.

**Figure 3:**
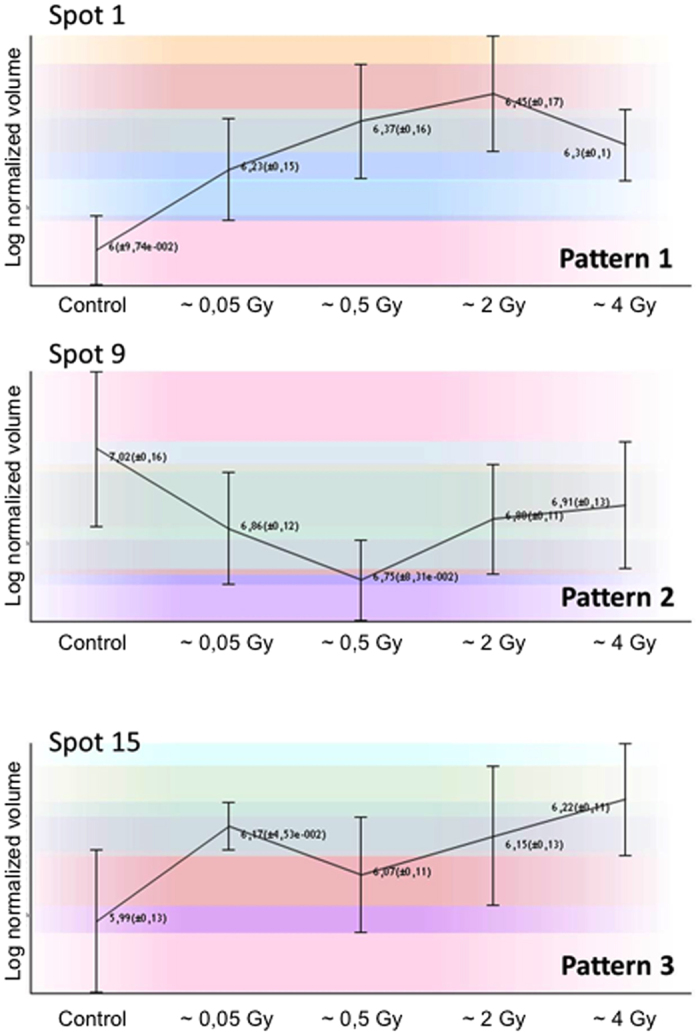
Characteristic patterns of spots differentially expressed upon [^3^H]thymidine incorporation. Each of these three patterns is related to the corresponding spot as presented in table 1 and supplementary data S1 and S2. The values correspond at least to three experiments.

From the 928 spots detected by 2D-gel electrophoresis and compared between experimental conditions, 3.6% were selected according to significant expression differences between treatments (Fig. 2) and were analyzed by mass spectrometry. A set of 44 proteins was identified and organized according to three characteristic patterns of expression profile (Fig. 3, Table 1 and supplementary data S1 and S2). Pattern 1 corresponds to the 24 first spots that exhibit a minimum amount in the control sample and a constant increase with [^3^H]thymidine incorporation until a plateau at 2 Gy. Pattern 2 corresponds to 2 spots displaying a V profile with a minimum amount at 0.5 Gy and a maximum amount with the control sample and/or the highest doses of [^3^H]thymidine. Pattern 3 corresponds to 8 spots displaying a minimum amount with the control sample and then a plateau with the different doses of [^3^H]thymidine. These patterns were assigned according to the three first correlation groups proposed by the statistical analysis by the Progenesis Samespots software based on similarity in expression profiles. Most of the selected spots displayed a minimum of intensity with the control sample, whereas protein amounts increased upon [^3^H]thymidine incorporation. Indeed, only 2 spots (6%) were observed at maximum levels in the control sample (pattern 2).

It is interesting to observe that all of the proteins implicated in the response to DNA damage or associated with oxidoreductase and/or antioxidant activity correspond to spots with lowest levels in the control sample and an increased expression in [^3^H]thymidine-treated samples. Globally, the proteins identified by mass spectrometry and potentially involved in the response of CHO cells to [^3^H]thymidine incorporation were associated with several biological processes. According to the “Gene Ontology” research tools of the Uniprot database (uniprot.org), 80% of the identified proteins were associated with metabolic processes, and 33% were involved in responses to stimulus (supplementary S2). More precisely, two proteins were described as implicated in the response to DNA damage (spot 12, Ubiquitin-conjugating enzyme E2 and spot 19, MICOS complex subunit Mic25). Approximately 44% of the identified proteins were associated with a biological regulation process, and four proteins displayed an oxidoreductase activity potentially involved in oxidative stress regulation (spot 13, Dihydropteridine reductase; spot 28, Isocitrate dehydrogenase; spot 44, Succinate dehydrogenase) or antioxidant activity (spot 41, Peroxiredoxin-4). The 2 spots belonging to pattern 2 were involved in glycolytic processes (spot 9, transketolase; spot 40, Fructose-bisphosphate aldolase A and Succinyl-CoA ligase).

Collectively, this proteomic analysis pointed to a general cell response to internal self-irradiation, which is detectable even at low doses. Importantly, this cell response acts in a nonlinear mode according to the doses.

### Low doses of incorporated [^3^H]thymidine induce mutagenesis

**Figure 4:**
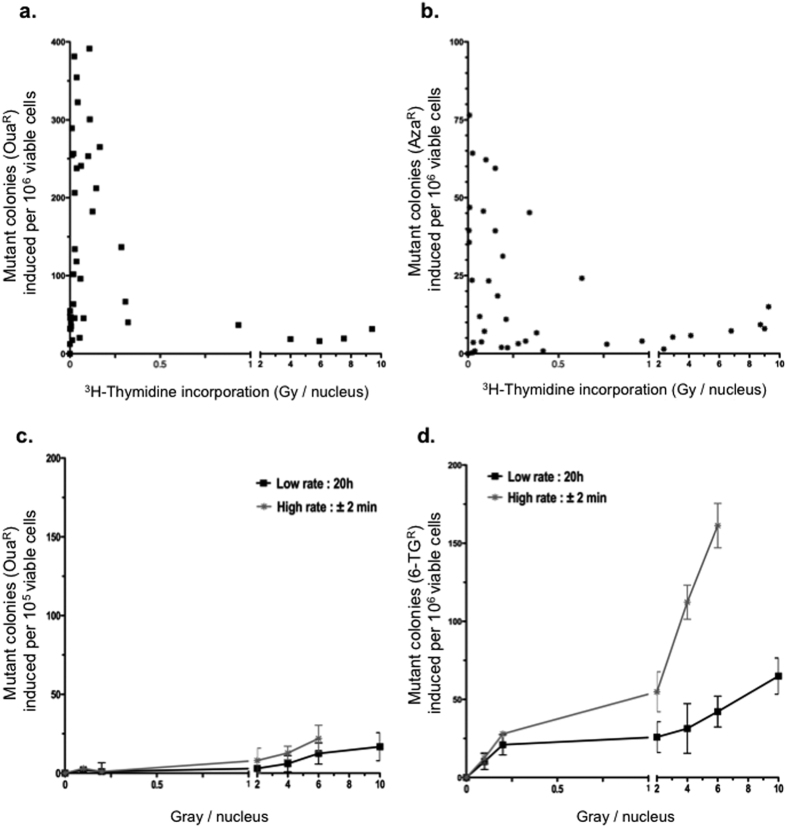
Mutagenesis induced by [^3^H]thymidine incorporation. (**a)** Mutant colonies induced by [^3^H]thymidine incorporation as indicated by viable ouabain-resistant colonies (locus *Na*^+^/*K*^+^ pump ATPase). (**b)** Mutant colonies induced by [^3^H]thymidine incorporation as indicated by viable 8-azaadenine-resistant colonies (locus APRT). Scatter plots contain combined data from at least three independent experiments. (**c)** Mutant colonies induced by ^60^Co external γ radiation with doses delivered as indicated by viable ouabain-resistant colonies (locus *Na*^+^/*K*^+^ pump ATPase). (**d)** Mutant colonies induced by ^60^Co external γ radiation with doses delivered as indicated by viable 6-thioguanine-resistant colonies (locus HPRT).

We measured mutagenesis at two loci: the *Na*^*+*^*/K*^*+*^ *-ATPase* membrane pump gene (Fig. 4a) and the *adenine phosphoribosyltransferase* gene (Fig. 4b). Inactivation of these genes leads to resistance to ouabain (Oua^r^) and 8-azaadénine (Aza^r^), respectively. Strikingly, instead of exhibiting a classical dose-response curve shape, a pronounced peak of Oua^r^ mutagenesis stimulation was induced at low [^3^H]thymidine doses between 0.05 and 0.5 Gy/nucleus. Mutagenesis then rapidly decreased at higher doses, reaching values close to zero at 1.5 Gy/nucleus (Fig. 4a). Higher doses going from 4 to 10 Gy/nucleus exhibited a modest dose-dependent induction. Importantly, this particular mutagenesis induction pattern was also observed for the *adenine phosphoribosyltransferase* (Aza^r^) gene using comparable doses of [^3^H]thymidine incorporation (Fig. 4b). Indeed, at this locus, [^3^H]thymidine incorporation also stimulated mutagenesis at doses between 0.05 and 0.5 Gy/nucleus. Then, mutagenesis decreased to reach a plateau for higher doses (from 1 to 10 Gy/nucleus). On the contrary, analysis of the mutagenesis of *Na*^*+*^*/K*^*+*^*-ATPase* membrane pump gene (Oua^r^) after exposure to external radiation (^60^Co γ–rays) at comparable dose rate (dose delivered in 20 h) or flash dose rate (dose delivered in 2 min) did not show any increase at low doses and a slight but significant increase for higher doses (Fig. 4c). In order to confirm this result, we analyzed mutagenesis of hypoxanthine-guanine phosphoribosyl transferase locus (hprt) leading to 6-thioguanine resistance (6-TG^R^). In this condition, we scored a fair induction of mutagenesis induced by external radiation at high doses for low dose rate. High dose rate show a stronger induction of mutagenesis (Fig. 4d).

### Mutation spectrum analysis

The mutagenesis spectra were then analyzed for two ranges of doses. The first range was defined for doses from 0.05 to 0.5 Gy/nucleus that corresponded to the mutagenesis peak and were denoted as low doses. The second range was defined for doses from 2 to 8 Gy/nucleus, corresponding to the mutagenesis plateau, denoted as high doses (see Fig. 4).

**Figure 5:**
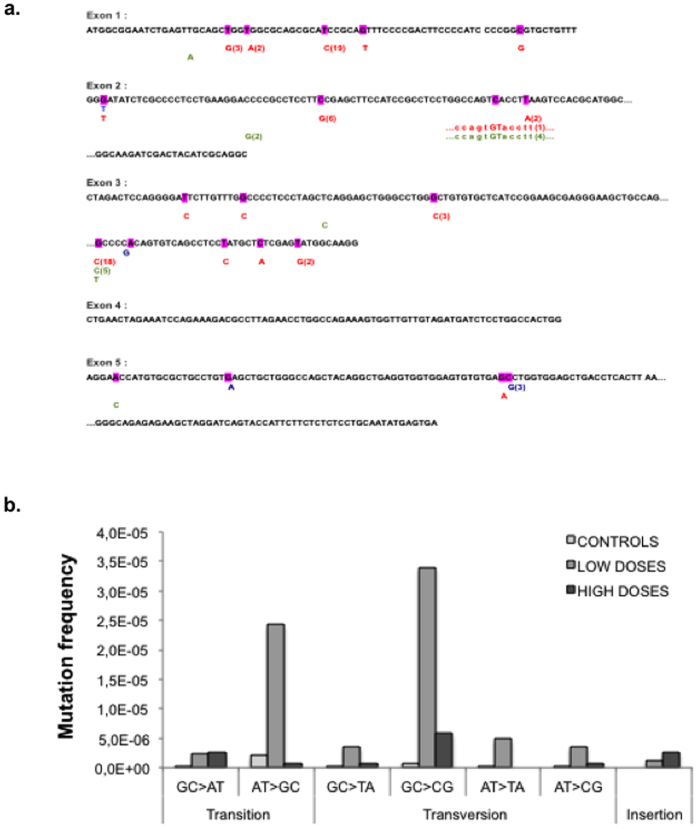
Mutagenesis analysis. (**a)** APRT locus sequence localization of mutations in control (blue) or upon low-dose (red) or high-dose (green) [^3^H]thymidine incorporation. Number in brackets designates the summation of individual clones for each mutation scored. (**b)** Types and frequency of mutations in control (black) or upon low-dose (light grey) or high-dose (dark grey) [^3^H]thymidine incorporation. Up to 110 independent *aprt*^−^ mutants clones were isolated and amplified for each condition (control, low- and high-doses).

We used the *aprt* gene locus because it has been extensively used for genomic mutagenesis analysis^7^. Up to 110 independent *aprt*^*−*^ mutants clones induced by [^3^H]thymidine incorporation were isolated and amplified for each condition (control, low- and high-doses). The entire coding sequence of the 5 exons of the *aprt* gene was sequenced (Fig. 5). Mutation analysis revealed that most of the mutations were base substitutions distributed all along the exons (Fig. 5a). No particular hot spot induced by [^3^H]thymidine incorporation was detected. The induced mutations revealed a signature of mutagenesis for low doses of [^3^H]thymidine incorporation, compared to control and to high doses (Fig. 5b). Indeed, at low doses, while all classes of base substitutions increased there was a strong stimulation of GC > CG transversion and AT > GC transition events (Fig. 5b).

### Oxidative stress is involved in mutagenesis induction by [^3^H]thymidine incorporation

**Figure 6:**
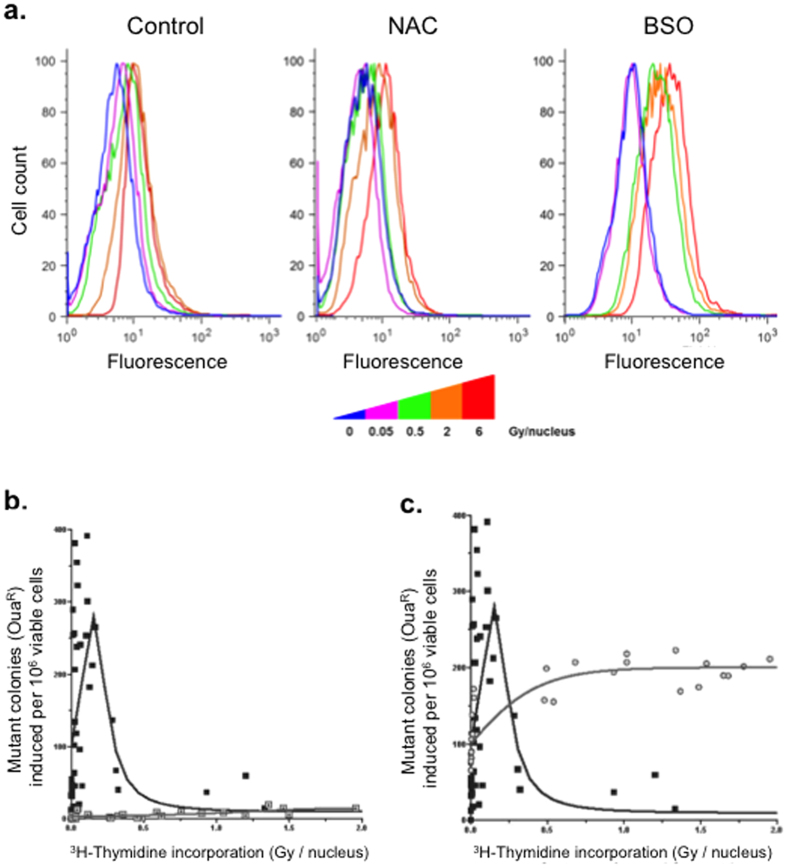
Mutagenesis is induced by oxidative stress from [^3^H]thymidine incorporation. (**a)** Intracellular ROS monitored using carboxy-H2DCFDA fluorescent quantification (flow cytometry) as an oxidative stress probe in control and 4 representative doses on each graph (0, 2 low doses and 2 high doses). (**b)** The effect of treatment with NAC (20 μM, 1 h prior [^3^H]thymidine incorporation) on the induction of mutant colonies by [^3^H]thymidine incorporation (ouabain resistance). Black squares and lines, control mutagenesis induced by [^3^H]thymidine incorporation (see Fig. 4A). Grey squares and lines, mutagenesis induced by [^3^H]thymidine incorporation after NAC treatment. (**c)** The effect of treatment with BSO (5 μM, 12 h prior [^3^H]thymidine incorporation) on the induction of mutant colonies by [^3^H]thymidine incorporation (ouabain resistance). Black squares and lines, control mutagenesis induced by [^3^H]thymidine incorporation (see Fig. 4A). Grey circles and lines, mutagenesis induced by [^3^H]thymidine incorporation after BSO treatment. Scatter plots present combined data from at least three independent experiments.

Given that radiation induces water radiolysis, we assessed whether mutagenesis could be attributed to an increase in ROS production by [^3^H]thymidine incorporation. Thus, [^3^H]thymidine cell cultures were assayed for ROS levels by flow cytometry using the carboxy-H2DCFDA dye (Fig. 6a). We analyzed the same range of doses used for mutagenesis analysis in this experiment (from 0.05 to 0.5 Gy/nucleus for low doses; from 2 to 8 Gy/nucleus for high doses). Fluorescence analysis showed a significant (*p* < *0.001*) increase of intracellular ROS in cells treated with [^3^H]thymidine (low and high doses) compared with untreated cells (Fig. 6a, left panel). Moreover, treatment of cells with the anti-oxidant N-acetyl-cysteine (NAC) prior exposure to [^3^H]thymidine abolished the ROS produced at low doses, whereas a significant increase (*p* < *0.001*) of intracellular ROS was maintained at high doses (Fig. 6a, middle panel). In this condition, the level of ROS induced by low doses did not significantly different from that observed in untreated cells, demonstrating that NAC is able to neutralize the ROS induced by low doses of incorporated [^3^H]thymidine. In contrast, L-buthionine sulfoximine (BSO) inhibits cellular defenses against ROS by interfering with glutathione (GSH) synthesis. Cells treated with BSO prior to exposure to [^3^H]thymidine exhibited a significant increase (*p* < *0.001*) in ROS for both, low and high doses (Fig. 6a, right panel), with the exception of the lowest doses analyzed (0.049 Gy/nucleus).

^3^H emits a 0.019 MeV beta-particle, yielding an average track of 1 μm (max: 6 μm) and 68% energy deposition in the cell nucleus^8^. Therefore, we hypothesized that [^3^H]thymidine incorporation may induce oxidative stress by ROS production within the nucleus. We thus measured the impact of NAC treatment on ^3^H]thymidine-induced mutagenesis (Oua^r^) (Fig. 6b). Interestingly, incubation with NAC prior exposure to [^3^H]thymidine significantly prevented mutagenesis (*p* < *0.0001*), and this effect was particularly evident at low doses (<0.5 Gy/nucleus) (Fig. 6b, grey square). This result shows that incorporation of [^3^H]thymidine induces mutagenesis via oxidative stress.

Interestingly, upon incubation with BSO prior to [^3^H]thymidine exposure, mutagenesis plateaued at higher doses (>0.5 Gy/nucleus) instead of decreasing as in sole [^3^H]thymidine exposure (Fig. 6c, grey circle). Taken together, these data suggest that ROS detoxification/protection mechanisms, such as GSH, are induced at high but not low doses of [^3^H]thymidine incorporation. Thus, stress below a certain threshold escapes cellular surveillance, thus promoting mutagenesis.

### APE-1 activity is induced by [^3^H]thymidine incorporation

**Figure 7:**
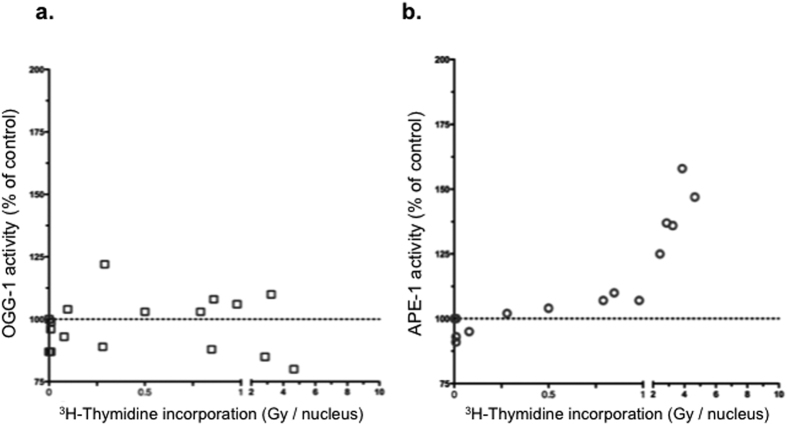
BER activities. (**a)** hOGG-1 activity. (**b)** APE-1 activity. Grey arrow denotes low-dose range. Scatter plots present combined data from at least three independent experiments. Grey arrows indicate low-dose range. APE-1 activity increase is significantly linked to [^3^H]thymidine incorporation (rs = 0.9241 ; p < 0.0001).

A major product of ROS interaction with DNA is the premutagenic lesion 7,8-dihydro-8-oxoguanine (8-oxoG). This lesion is repaired via the base excision repair (BER) pathway, which is initiated in mammals by the DNA glycosylase OGG1 to generate apurinic/apyrimidinic (AP) sites^9^. We therefore measured the OGG1 activity in the cell extracts and found that it did not vary significantly within the range of doses used (Fig. 7a).

Importantly, repair of AP sites is carried out by APE-1, the major AP endonuclease in mammalian cells, accounting for greater than 95% of the total AP site incision activity (10). Interestingly, APE-1 activity was significantly induced by high doses but not by low doses of incorporated [^3^H]thymidine (Fig. 7b), suggesting the existence of a threshold of stress for the specific induction of APE1 activity that is essential for DNA repair of all kind of oxidized DNA.

## Discussion

Endogenous stress is a major source of genomic alteration potentially leading to spontaneous tumor or senescence initiation; however, the process is *per se* difficult to detect and analyze. Both replication stress and oxidative stress are classically evoked at the initiation of tumorigenesis and senescence^10^,^11^,^12^. Remarkably, these two kinds of stress are linked. Indeed, endogenous oxidative stress generates a replication stress^13^, underlying the importance of endogenous ROS in genome instability and cell fate.

Here, we exposed cells to a radioactive compound, in order to generate *in fine* an internal-like stress. Following incorporation into the DNA, the biological consequences and cell responses clearly differ from exposure to exogenous radiation and actually result from *in situ* auto-irradiation, i.e. an endogenous-like stress. Consequently, part of the response should be representative of the endogenous stress. First, the production of ROS by the cell metabolism appears to be an essential feature, as state above. Incorporation of [^3^H]thymidine can generate ROS through water radiolysis, and we show here that incorporation of [^3^H]thymidine actually increases the level of endogenous ROS. Second, cellular responses to endogenous *versus* exogenous sources of genotoxic stress differ, and depend on the energy of the radioactive compound. Indeed, we previously demonstrated that incorporation of tritium, which emits a low energy (0.019 MeV) into DNA, provokes more DNA breaks and homologous recombination events than does ^14^C, which emits 8-fold higher energy levels (0.157 MeV)^8^. This difference can be explained by the fact that low energy radiation is locally concentrated, and consequently, 70% of the tritium energy is deposited into the nucleus, whereas ^14^C radiation can go beyond the nucleus and deposit its energy anywhere in the cell, with only 20% of the energy deposited into the nucleus. Importantly, at the same doses, exposure to external γ-rays had no impact on DNA beaks and homologous recombination^8^. Here, exposure to exogenous radiation does not generate mutagenesis at low dose even at low rate exposure, in contrast with incorporation of [^3^H]thymidine (see Fig. 4). These data underlines the fact that the phenomena described here actually result from the cell response to endogenous stress. Therefore, incorporation of [^3^H]thymidine represents a tractable tool to monitor the cellular response to an endogenous source of stress targeted to the nucleus, i.e. the genome.

The data presented herein indicate that incorporation of [^3^H]thymidine into DNA induces a general regulation of the cellular REDOX status and the DNA damage response, as described by the mutagenesis analysis, in association with the assessment of APE1 and GSH activities. Importantly, the proteomic analysis confirms these conclusions, at a global level. Consistent with these findings, exposure of mammalian cells to various alkylating carcinogenic agents produces a nonlinear dose response. However, in that case and in contrast with the present data, no biological effects were detected at low doses^14^,^15^,^16^. Here, upon [^3^H]thymidine incorporation into the cellular DNA, we detect mutagenesis at low, non-toxic doses, reaching a peak at doses below 0.2 Gy/nucleus, then decreasing. Together, the data presented here demonstrate that below a threshold of endogenous DNA irradiation, the stress escapes cellular surveillance, thus permitting mutagenesis.

Importantly, we identified the type of [^3^H]thymidine-induced mutagenic stress as being an oxidative stress. This result was demonstrated by the production of intracellular ROS using the fluorescent probe and by the fact that the anti-oxidant NAC suppresses the induction of mutagenesis. In addition, the mutagenesis spectrum of point mutations is compatible with oxidative stress-induced mutagenesis. Indeed, the strong increase in GC to CG transversions is a signature of oxidative damage by singlet oxygen^17^,^18^,^19^. In particular, the oxidation of 8-oxoguanine can form imidazolone, guanidinohydantoin or spiroiiminodihydantoin, which could be responsible for GC to CG transversions^20^. The explanation for increase in AT to GC transitions is less obvious, but it could be due to the oxidation of the adenine opposite [^3^H]thymidine. Indeed, 8-hydroxyadenine and 2-hydroxyadenine are both formed by ionizing radiation in cells^21^,^22^,^23^. The former has been shown to yield AT to GC transitions^24^ and 2-hydroxyadenine can stably pair with cytosine in DNA and induce AT to GC mutations in mammalian cells^25^,^26^. Furthermore, we identified various cell responses to the endogenous stress used here. Indeed, at high doses, the inhibition of GSH production by BSO abolished the decrease in mutagenesis, demonstrating that free radical detoxifying systems are activated with increasing doses; however, a threshold should be reached. In addition, the induction of APE-1 enzymatic activity confirms the participation in the cellular response to ROS of enzymes involved in the repair of oxidative DNA damage. Remarkably, while OGG1 activity is not modified by the irradiation, APE-1 activity is induced at high but not at low doses requiring a threshold of stress comparable to that required for ROS detoxification systems. APE1 was shown to protect cells from the consequences of irradiation by its involvement in the repair of both, oxidized bases and abasic sites^27^,^28^. Therefore specific targeted regulation of APE1 activity allows to control DNA repair of all type of oxidative DNA damages, in contrast with enzymes involved in the repair of specific damages -such as OGG1 whose activity does not significantly vary, accounting thus for the modest effect on GC > AT transversions. The association of the induction of detoxifying systems and DNA repair enzymes provides an explanation for the decreased mutagenesis at high doses and underlines the existence of a threshold for their induction.

**Figure 8:**
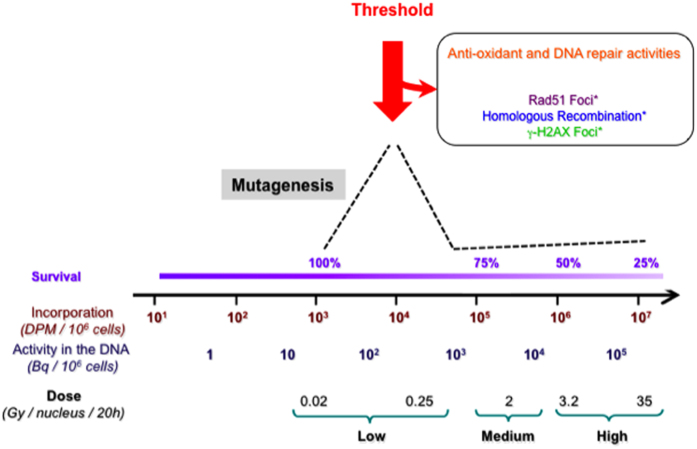
Threshold model for cell response to stress from endogenous source. A threshold of stress (red arrow) from endogenous source is needed to activate detoxification and DNA repair of oxidative damages. Note this threshold of dose is also required to generate γH2AX foci and to induce homologous recombination^1^. Below this threshold detoxification and DNA repair are not activated leading to mutagenesis (black dotted line) induced by the oxidative stress. Dotted line: mutagenesis. *from^1^.

These data can be summarized in the model of Fig. 8. In such a model, below 0.2 Gy/nucleus, the induced stress escapes cell surveillance, thereby allowing the ROS produced to attack the DNA without activation of the BER. At higher doses, the stress triggers the cellular response, imcluding both detoxifying the ROS produced and repairing oxidative DNA damage. This effect reduces mutagenesis and results in a nonlinear dose-response curve shape that exhibits a peak.

The use of both natural and synthetic radioactive compounds raises many environmental concerns. In the case of external exposure, the energy emitted should be sufficient for radiation to reach cellular targets. Such kind of exposure can result from medical exposure, industrial catastrophe or use of atomic weapons. These events are rare or out of ordinary. Contamination with radioactive compound can result from pollution from nuclear industry, representing thus a permanent risk. Upon incorporation into cells and tissues, contaminations with radioactive compounds cause *in situ* self-irradiation, therefore close to important biological targets. Moreover, the low penetration becomes an amplifying factor as energy is focused in the immediate vicinity of the incorporated isotope, concentrating thus the energy deposition. Here, we show that low doses of tritium into the DNA induce more mutagenesis than higher doses because low doses escape cellular surveillance, while, in contrast, higher doses trigger a cellular response. Noteworthy, these low doses have little (or no) effect on cell viability^1^,^2^. The absence of cell toxicity constitutes an additional danger given that the survival of cells carrying genetic alterations increases oncogenic risk or allows for transgenerational transmission of such genetic alterations. Therefore, these data lead to the apparent paradox: low doses of a compound emitting internal low energy, lead to pernicious increased endogenous *in situ* stress without significantly affecting cell viability, thus increasing the risks of generating viable cells with genomic instability.

## Methods

All methods were carried out in accordance with the relevant guidelines. All experimental protocols were approved by CNRS and the Commissariat à l’Energie Atomique et aux Energies Alternatives.

### Cell lines

We used two Chinese hamster cell lines (CHO): CHO-DRA10 and CHO-AT3-2 for mutagenesis analysis of Na^+^/K+ pump ATPase and adenine phosphoribosyltransferase (APRT) loci. Cells were maintained at 37 °C with 95% air/5% CO2 in Dulbecco’s modified Eagle’s medium (DMEM) supplemented with 10% fetal bovine serum (FBS). CHO-DRA10 cells were a generous gift from Dr. M. Jasin (Memorial Sloan Ketterin Cancer Center, NYC, USA) and CHO-AT3-2 cells were a generous gift from Dr. E. Sage (Institut Curie, Orsay, France). FBS was commercialy FBS (USDA approved) from Biological Indutries (Kibbutz Beit-Haemek, 25115, Israel) and certified by the International Serum Industry Association.

For oxidative stress analysis, cells were incubated with N-acetyl cysteine (NAC; 20 μM, 1 h 00 prior [^3^H]thymidine incorporation) to increase free radical cell detoxification. For the contrasting effect, cells were incubated with L-buthionine sulfoximine, a specific inhibitor of the glutathione synthesis pathway (BSO; 5 μM, 12 h 00 prior [^3^H]thymidine incorporation), to poison cellular defenses against reactive oxygen species (ROS).

### Radioactive labeling of cells

[Methyl-^3^H]TdR (cat # TRK 637; 45,0 Ci/mmol) purchased from Amersham (GE Healthcare Europe, Orsay, France) was diluted in regular medium. Cells were treated in exponential and asynchronous growth at the same concentration (0.1 mM) but at various specific activities (0.05 to 5 μCi/ml). Cells were incubated for 20 h at 37 °C with 5% CO_2_; at this time, incorporation plateaued, and greater than 95% of cells contained labeled nucleotides. The radioactive medium was subsequently discarded, and cells were washed twice in phosphate-buffered saline (PBS) before processing^1^. Experiments were repeated at least thrice.

### External radiation exposure

Cells were irradiated in exponential and asynchronous growth with a medical ^60^Co γ-rays external source. Each dose was delivered either on 2 minutes (high rate) or 20 hours (Low rate) in an specific incubator (37 °C with 5% CO_2_). Dosimetry was evaluated inside the incubator.

### Protein extraction

Cells (10 to 20.10^6^ cells) were washed in PBS, and the cell pellet was re-suspended in lysis buffer (20 mM HEPES, 1 mM DTT, 0.5% NP40, a protease inhibitor cocktail (complete mini, Roche), and a phosphatase inhibitor cocktail (Sigma)). Following 20 min at 4 °C, the suspension was centrifuged at 10000 × *g* for 10 min. The pellet containing cell nuclei was homogenized in solubilization buffer (9 M urea, 4% CHAPS, 0.05% Triton X100, 65 mM DTT and protease inhibitor cocktail). Proteins were then solubilized and quantified as previously described^29^.

### Two-dimensional electrophoresis

Two-dimensional electrophoresis was performed with independent biological experiments and at least three replicates using precast 18-cm strips at pH range 6 to 11 (GE Healthcare) for the first dimension and 12% acrylamide SDS-polyacrylamide gel for the second dimension^5^,^30^. Analytical gels were performed with 100 μg of protein and stained with Lava-purple (Serva) as recommended by the manufacturer and scanned to images with a Typhoon 9400 (GE). A preparative gel was performed for each condition with 250 μg of protein and stained using a MS compatible silver staining protocol^31^.

### Image analysis

Images from analytical gels were analyzed using the Samespots software v4.0 (Non-linear Dynamics, UK). Gel replicates were grouped to create a global analysis with all conditions. Spots of each samples were compared between control and irradiated conditions. A multivariate statistical analysis was performed using the statistic mode of the Samespots software (Non-linear Dynamics, UK). Spots with significant differences (ANOVA t-test p < 0.05) were first chosen. Then, only spots with a q-value <0.05 and a power >0.8 were finally selected. Spots of interest were selected for subsequent protein identification by mass spectrometry analysis and were picked up using the corresponding preparative silver stained gels.

### LC MS/MS analysis

Spots were washed with 300 μL of water and then 300 μL of 25 mM NH_4_HCO_3_. Destaining was performed twice in the presence of 300 μL of 50% acetonitrile in 25 mM NH_4_HCO_3_. Gel pieces were then dehydrated twice by 300 μL of 100% CH_3_CN and finally dried at 37 °C for 1 h. Eight microliters of a trypsin solution (Sequencing Grade Modified Trypsin, Promega, Madison, USA) at a concentration of 0.0125 μg/μL in 25 mM NH_4_HCO_3_ was added to every spot. Digestion was performed overnight at 37 °C and was stopped by addition of 2 μL of 2% formic acid. Digests were sonicated in an ultrasonic bath for 10 minutes and supernatants were transferred into HPLC polypropylene tubes. The protein digests were analysed using a Q-TOF mass spectrometer (Maxis ; Bruker Daltonik GmbH, Bremen, Germany), coupled to a nano-chromatography system (HPLC 1200, Agilent Technologies, Santa Clara, CA, USA) interfaced with an HPLC-Chip system (Chip Cube, Agilent Technologies). Samples were concentrated onto the online pre-column (Agilent Technologies, part G4240-62006, Zorbax 300SB-C18, 40 nL, 5 μm particle size) at a flow rate of 4 μL/min using 0.1% formic acid. After pre-concentration, peptides were separated with a reversed-phase capillary column (Agilent, Zorbax 300SB-C18, 0.075 × 150 mm) at a flow rate of 0.3 μL/min using a 2 steps gradient (3% to 27% acetonitrile in 14 min then 27% to 72% in 5 min), and eluted directly into the mass spectrometer. The instrument was operated in the positive ion mode with a capillary voltage of 1.8–2.1 kV, and a dry gas flow rate of 4 L/min at 140 °C. After an initial MS scan at 2 Hz, an information-dependent acquisition (IDA) mode was employed over the mass range of 300–1400 Th, for the five most intense precursors with an ion intensity above 2000 counts, a charge state of (+2) or (+3) and an active exclusion of 0.15 min after 2 spectra. Collision energies were set to automatically adjust according to the charge state and mass of the precursor ions. Proteins were identified by MS/MS by information-dependent acquisition of fragmentation spectra of multiple charged peptides. Up to 5 data-dependent MS/MS spectra were acquired in positive ion mode. MS/MS raw data were analysed using Compass DataAnalysis software v.4.0.275.0 (Bruker Daltonik GmbH, Bremen, Germany) to generate the peak lists. The Swissprot database (release 2015_03, 547 964 sequences) was queried locally using the Mascot search engine v.2.4.0 (Matrix Science, http//www.matrixscience.com) and with the following parameters: Rodentia for the taxonomy, trypsin as enzyme, 1 missed cleavage allowed, carbamidomethylation of Cystein as fixed modification, oxidation of Methionine as variable modification. Mass tolerance was set to 10 ppm on full scans and 0.05 Da for fragment ions. Proteins were validated once they contained at least two peptide with a p value <0.05. When identified, contaminants proteins (such as keratine) were removed from the identification list and only the two top hit remaining proteins with similar scores and emPAI were considered. The mass spectrometry proteomics data have been deposited to the ProteomeXchange Consortium (http://proteomecentral.proteomexchange.org) via the PRIDE partner repository^32^ with the dataset identifier <PXD003542>.

### Mutagenesis measurements

Cells were trypsinized, counted and divided into three fractions. The first fraction was used to measure the incorporation of labeled nucleotides. The second was used to measure the colony-forming efficiency, and the third was plated with 3 mM of ouabain (Sigma-Aldrich) or 0.4 mM 8-azaadenine to measure the *Na*^*+*^*/K*^*+*^ *pump ATPase* and APRT mutagenesis frequency, respectively^7^,^33^,^34^.

### Measurement of labeled nucleotides incorporated into DNA

Cells were suspended and DNA was precipitated in ice-cold 10% trichloroacetic acid (TCA). Precipitated DNA was recovered on GF/C filters (Whatman), which were subsequently washed with 10% and 5% TCA. Incorporation of [^3^H]-thymidine into DNA was determined by subjecting the filters to liquid scintillation counting. We expressed the incorporation rate as dpm (disintegrations per minute) per 10^6^ cells. We then estimated the dose delivered into each nucleus using converting factors described elsewhere^8^. For ^3^H incorporation into the nucleus, the converting factor is 1 disintegration = 2.70 10^−3^ Gy. The doses were then calculated for 20 h incorporation.

### Molecular analysis of mutants

Following a 6-day phenotypic expression period, clonally independent CHO-AT3-2 *aprt*^*−*^ mutants induced by [^3^H]-thymidine were isolated by phenotypic selection using the base analogue 8-azaadenine according to established protocols^7^. Each clone was grown into separate plates to 10^6^ cells. Genomic DNA was extract from harvested cells using the Nucleospin kit (Macherey Nagel, Düren, Deutschland). Mutant *aprt* alleles were amplified by PCR and then sequenced by GATC Biotech, France. Wild type *aprt* sequences from our cell line served as the control. Mutagenesis analysis was performed with Clustalw software.

### Oxidative stress probe

Immediately after tritium contamination, cells were washed in PBS three times, harvested and nucleofected with a 5-(and-6)-carboxy-2′,7′-dichlorodihydrofluorescein diacetate probe (carboxy-H2DCFDA) provided by Invitrogen (Life Technology, Cergy Pontoise, France). Nucleofection was optimized from Amaxa protocols for CHO cell lines and performed with Amaxa Nucleofector II Device (Lonza, France). Nucleofected cells were then analyzed by flow cytometry (FACScalibur, Becton-Dickinson) to measure fluorescence. Statistical analyses were performed with FlowJo software (Tree Star, Inc. Ashland, OR, USA).

### Enzymatic activities

Contaminated and control cells were washed with PBS and trypsinized. The numbers of live and dead cells were determined by counting after trypan blue staining. After two additional washes with PBS, cells were stored as dried pellets at −80 °C until extraction. Extracts were obtained by sonication of pellets in 20 mM Tris–HCl, pH 7.5; 250 mM NaCl; 1 mM EDTA containing a cocktail of apoprotinin, antipain, and leupeptin (0.8 μg/μl each). The homogenate was centrifuged at 20,000 × *g* for 30 min at 4 °C, and aliquots of the supernatant were stored at −80 °C for biochemical assays. Protein concentrations were measured using a Bio-Rad assay kit (Bio-Rad Laboratories, Richmond, CA) with bovine serum albumin as a standard.

#### 8-oxoG DNA glycosylase assay

A 34-mer oligonucleotide containing an 8-oxoG at position 16 and labeled at the 5′ end with Cy5 was hybridized to its complementary oligonucleotide containing a cytosine opposite the lesion yielding the 8-oxoG:C duplex. In a standard reaction mixture, protein extracts (4 μl final volume) were added to a 10 μl reaction mixture containing 150 fmoles of the 8-oxoG:C labeled duplex in 20 mM Tris–HCl pH 7.1, 1 mM EDTA, 100 mM NaCl, 1 mg/ml BSA and 5% glycerol. The reaction mixtures were incubated at 37 °C for 1 h. NaOH (0.1N final concentration) was added, and the mixture was further incubated for 15 min at 37 °C and stopped by adding 4 μl of formamide dye, followed by heating for 5 min at 95 C. The products of the reaction were resolved by denaturating 7 M urea −20% polyacrylamide gel electrophoresis. Gels were scanned and band intensities were quantified using a Storm PhosphoImager (Amersham Bioscience).

#### AP site (apurinic/apyrimidinic site) incision assay

A 34-mer oligonucleotide containing a single tetrahydrofuranyl artificial AP site at position 16 and labeled at the 5′ end with Cy5 was hybridized to its complementary oligonucleotide containing a cytosine opposite the lesion. In a standard reaction mixture, protein extracts (4 μl final volume) were added to a 10-μl reaction mixture containing 150 fmoles of the THF oligonucleotide labeled duplex in 25 mM Tris–HCl pH 7.1, 1 mM MgCl_2_, 1 mg/ml BSA and 5% glycerol. The reaction mixtures were incubated at 37 °C for 30 min. The reaction was stopped by adding 4 μl of formamide dye followed by heating for 5 min at 95 °C. The products of the reaction were resolved by denaturating 7 M urea/20% polyacrylamide gel electrophoresis. Gels were scanned, and band intensities were quantified using a Storm PhosphoImager (Amersham Bioscience).

### Statistical analysis

In contrast to external radiation as photon radiation, nucleotide incorporation varies among repeated experiments. As a consequence, averages and standard deviations cannot be calculated and plotted; all data were graphed as scatter plots. Then, we calculated the Spearman’s Rho rank correlation coefficient (rs) for the relationship between two non-normally distributed variables. This information was used for analysis of the nonlinear relationship between radioisotope cellular contamination and the biological end-points (mutagenesis, enzyme activities). All results demonstrated a significant Spearman’s Rho rank correlation coefficient. Regression curves were calculated using a Gaussian convolution data transformation of the non-linear distributions. All analyses were performed with Prism (GraphPad Software, Inc., San Diego, CA).

## Additional Information

**How to cite this article**: Saintigny, Y. *et al*. A threshold of endogenous stress is required to engage cellular response to protect against mutagenesis. *Sci. Rep.* **6**, 29412; doi: 10.1038/srep29412 (2016).

## Supplementary Material

Supplementary Information

Supplementary Table S1

Supplementary Table S2

## Figures and Tables

**Table 1:** The three groups of proteins according to characteristic patterns of expression.

Pattern	Protein	Accession	Spot	Fold change
1	Elongation factor 1-alpha 1	P10126	1	2,87
	PAI1 RNA-binding protein 1	Q6AXS5	1	2,87
	Succinyl-CoA ligase	P13086	2	1,56
	SUMO-conjugating enzyme UBC9	P63280	4	1,53
	Peptidyl-prolyl cis-trans isomerase A	P14851	4	1,53
	Peptidyl-prolyl cis-trans isomerase A	P14851	6	1,45
	U2 small nuclear ribonucleoprotein A′	P57784	7	1,83
	Phosphoserine aminotransferase	Q99K85	10	1,47
	Destrin	Q7M0E3	12	1,66
	Ubiquitin-conjugating enzyme E2 variant 1	Q9CZY3	12	1,66
	Dihydropteridine reductase	P11348	13	1,53
	Voltage-dependent anion-selective channel protein 2	Q60930	14	1,42
	ELAV-like protein 1	P70372	17	1,51
	Heterogeneous nuclear ribonucleoprotein A1	P09651	17	1,51
	Proteasome subunit beta type-1	O09061	18	1,70
	MICOS complex subunit Mic25	Q91VN4	19	1,66
	PINCH-1	Q99JW4	19	1,66
	Peptidyl-prolyl cis-trans isomerase A	P14851	20	1,30
	Citrate synthase	Q8VHF5	22	1,44
	Adenylate kinase 4	Q9WUS0	23	1,71
	Peptidyl-prolyl cis-trans isomerase B	P24368	25	1,52
	Phosphoglycerate kinase 1	P50310	26	1,34
	Galectin-3	P47953	27	1,50
	Isocitrate dehydrogenase	P54071	28	1,54
	Glutathione S-transferase Y1	Q00285	28	1,54
	Glutathione S-transferase P	P46424	31	1,36
	Peptidyl-prolyl cis-trans isomerase FKBP3	Q62446	33	1,30
	Voltage-dependent anion-selective channel protein 1	Q9Z2L0	34	1,47
	Snx3	Q9CSC2	35	1,41
	Succinate dehydrogenase	P21913	44	1,34
	Peptidyl-prolyl cis-trans isomerase FKBP3	Q62446	44	1,34
2	Transketolase	P40142	9	1,93
	Fructose-bisphosphate aldolase A	P05065	40	1,47
	Succinyl-CoA ligase [ADP/GDP-forming] subunit alpha	P13086	40	1,47
3	Peptidyl-prolyl cis-trans isomerase B	P24368	3	1,69
	Protein NipSnap homolog 3B	Q9CQE1	5	1,79
	Protein disulfide-isomerase A5	Q921X9	15	1,67
	TCP-1-zeta	P80317	15	1,67
	Proteasome subunit beta type-5	O55234	16	1,42
	Transketolase	P40142	32	1,58
	GTP-binding nuclear protein Ran	P62826	39	1,45
	Cytochrome b-c1 complex subunit Rieske	Q9CR68	41	1,66
	Peroxiredoxin-4	Q9Z0V5	41	1,66
	ATP synthase subunit alpha	Q03265	42	2,06

(see examples in Fig. 3 and details in supplementary data S1 and S2).
