# Supplementary material for: A threshold of endogenous stress is required to engage cellular response to protect against mutagenesis
Source: Sci Rep. 2016 Jul 11;6:29412. doi: 10.1038/srep29412 (PMC4942696; doi:10.1038/srep29412)
Supplement: Supplementary Information [file srep29412-s1.pdf]

A threshold of endogenous stress is required to engage cellular response to protect against mutagenesis

Yannick Saintigny, François Chevalier, Anne Bravard, Elodie Dardillac, David Laurent, Sonia Hem, Jordane Dépagne, J. Pablo Radicella and Bernard S. Lopez

Supplementary data

Supplementary data Table S1: Complete list of identified proteins, with their corresponding peptides, of spots differentially expressed following [3H]thymidine treatment.

Supplementary data Table S2: Uniprot analysis of protein ID identified by mass spectrometry: Gene ontology (GO), Gene ontology (biological process), Gene ontology (molecular function), Gene ontology (cellular component) and Pathway as obtained from the website (http://www.uniprot.org/uploadlists/).
